# Mental well-being and diversity, equity, and inclusiveness in the veterinary profession: Pathways to a more resilient profession

**DOI:** 10.3389/fvets.2022.888189

**Published:** 2022-07-29

**Authors:** Florentine Scilla Louise Timmenga, Wiebke Jansen, Patricia V. Turner, Nancy De Briyne

**Affiliations:** ^1^Faculty of Veterinary Medicine, Utrecht University, Utrecht, Netherlands; ^2^Federation of Veterinarians of Europe, Brussels, Belgium; ^3^Global Animal Welfare and Training, Wilmington, MA, United States; ^4^Department of Pathobiology, University of Guelph, Guelph, ON, Canada

**Keywords:** mental health, work-life balance, role models, inclusion, veterinarians, veterinary school, well-being, programs

## Abstract

Mental well-being (MWB) and diversity, equity, and inclusiveness (DEI) continue to be critical within the veterinary profession but there is less information regarding how professional associations around the world tackle these issues. A mixed-method study including an international online survey in English (*n* = 137 responses *via* snowball sampling), fourteen interviews, and two webinars was used to identify the availability and impact of MWB and DEI support programs for veterinarians. Survey results showed that more veterinary organizations designated MWB and DEI challenges (54%, *n* = 43/79 and 58%, *n* = 45/78, respectively) as a key priority area than veterinary clinics (26%, *n* = 15/57 and 33%, *n* = 19/57, respectively). Whereas, MWB support programs were available in a moderate number of mainly English-speaking countries, DEI support programs were available in only a few countries and focused primarily on specific groups, with an unknown impact due to their recent implementation. Universally, survey respondents believed activities for specific groups, such as MWB webinars, training, and awareness campaigns, as well as MWB/DEI helplines and DEI peer-to-peer support programs had a high impact (median 3.5–4/5) yet were underemployed by both veterinary organization and veterinary clinics. Further feedback from respondents during focused interviews indicated that requiring initial and continuing training as well as tailored group activities would be most beneficial to improve MWB/DEI throughout the veterinary professional career. There are many areas of the intersection between MWB and DEI that remain to be elucidated in the future studies. Having a sufficient sample size, improving accessibility, and addressing varying cultural perceptions are the main challenges, as seen in our study. To truly address MWB and DEI disparities, change is also needed in veterinary workplace culture and environment. In conclusion, raising awareness for an inclusive profession, including increasing openness and acceptance to enhance DEI and destigmatizing MWB challenges, is needed to ensure a thriving, modern veterinary profession.

## Introduction

The veterinary profession offers multifaceted and ambitious careers requiring highly flexible and resilient professionals. While recognizing the abundantly positive aspects of work within the different domains of the veterinary profession, the challenging socioeconomic and cultural working climate has been recognized as a source of veterinary mental well-being (MWB) issues. Veterinary MWB is a concept that was defined within organizations and research, e.g., applying the “Five Pillars of Health Framework” to describe good MWB and the sectional division of Body, Mind, Behavior, Context, and Spirit (1). In the last decade, numerous reports have identified important stressors in the veterinary profession, particularly from English-speaking countries (i.e., UK, USA, Canada, Australia, and New Zealand). These have included long working hours (2–5), lower income compared to other medical professionals (6), challenging client communications (3, 6–10), demanding work-life balance (6, 11), and high student debt (12, 13) and resulted in compassion fatigue (14, 15), burnout (14–16), veterinarians feeling they have a life not worth living (17), and other forms of stress (18). In particular, early career female practitioners generally seem to experience more negative stressors compared to more seasoned male colleagues (2, 13, 19). In the recent years, several studies reported that veterinary MWB has been under increasing pressure due to the global COVID-19 pandemic (20–24).

A lack of diversity, equity, and inclusiveness (DEI) in the veterinary profession is also frequently reported as a stressor in the UK and USA (25–29). Snyder et al. (30) reported that the veterinary profession in the USA was the least racially diverse (consisting of 93.8% White non-Hispanics) with one of the lowest proportions of people of color, compared to other health occupations. Globally, the number of female veterinarians is increasing and has outpaced male veterinarians in many countries and regions (31–38) with a total of 58% of European veterinarians being female (2). Despite this, a gender pay gap is commonly reported in veterinary practice (2, 4), as well as client sexism (39), a lack of respect for female practitioners following childbirth and/or work part-time (40, 41), and gender mismanagement and leadership visibility issues, with females rarely climbing to the upper veterinary hierarchy (34, 41, 42). Members of the Lesbian, Gay, Bisexual, Trans, Queer, and Intersex (LGBTQI+) community experience more mental health problems and suicidal ideation in veterinary school and as veterinary professionals (11, 43). A global survey by the International Veterinary Student Association (IVSA) concluded that student discrimination is a serious issue due to their ethnicity or sexual orientation at most veterinary universities (44).

**Figure 1:**
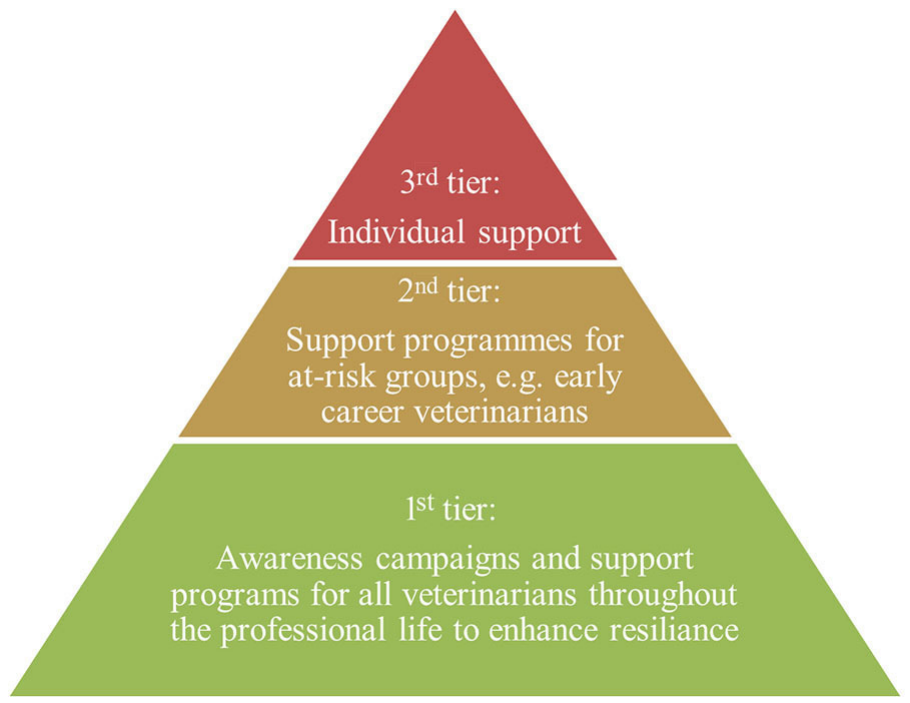
Holistic three-tiered approach of MWB support programs in veterinary medicine (adapted from Moir and Van den Brink (45)).

To address these challenges, many national and regional veterinary organizations have launched awareness campaigns and begun to implement MWB support programs using a holistic approach (Figure 1). Private organizations such as Mind Matters International [MMI; based within the Royal College of Veterinary Surgeons (RCVS)], Vetlife, and Not One More Vet have been established to raise awareness and improve MWB on an (inter) national level for individuals in need. Despite these programs, it is recognized that not all veterinarians receive the support they need due to local lack of availability and stigmatization of MWB issues (12, 46).

While recognizing the importance of the identification of MH- and DEI-related issues in the veterinary profession, it is crucial to move the focus to action and intervention. Apart from a few studies evaluating support programs on a national level (47–49), no research has investigated the design and impact of different support programs globally available for veterinarians including those targeting DEI. Therefore, this study aimed to (i) identify the availability, design, and impact of different MWB and DEI support programs and initiatives, as well as exploring possible contributing factors, and (ii) identifying potentially underexploited strategies for all three tiers to mitigate risk and enhance veterinary MWB and DEI.

## Materials and methods

**Figure 2:**
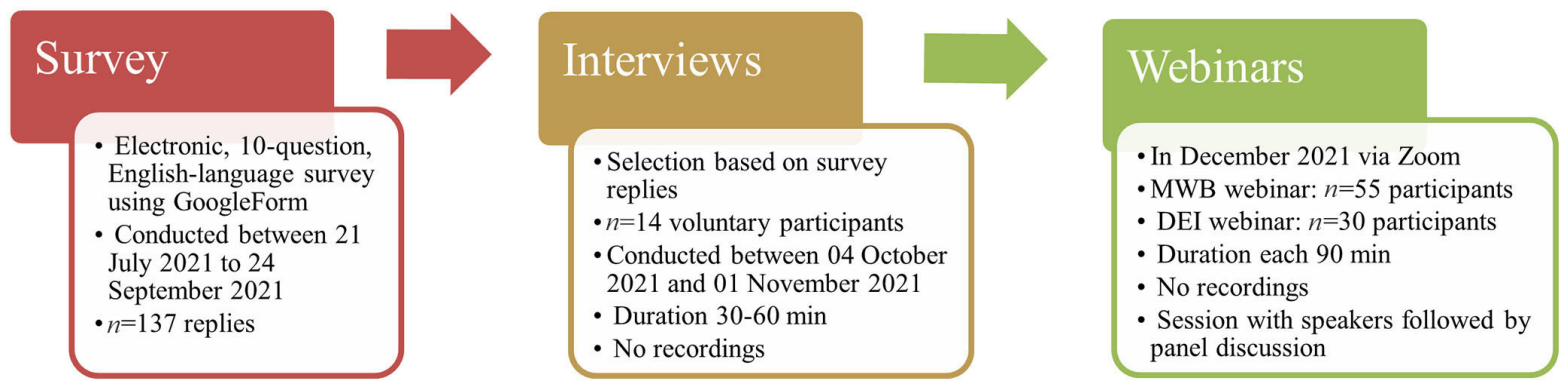
Timeline and participants of the three stages of the mixed-method study.

This mixed-methods study consisted of a quantitative cross-sectional online survey, interviews with a smaller sample group based on the survey outcomes, and input received following two webinars (Figure 2). The STROBE (Strengthening the Reporting of Observational Studies in Epidemiology) guideline for cross-sectional studies (104) and the Checklist for Reporting Results of Internet E-Surveys (CHERRIES) (105) were used for reporting (Supplementary Tables S1, S2).

### Survey

The survey and research protocol were developed by the Federation of Veterinarians of Europe (FVE) and approved by World Veterinary Association (WVA) and Zoetis in line with FVE privacy policy. Informal beta testing of the questionnaire was carried out within FVE. Targeted e-mails with an open link to the questionnaire on Google Forms were sent to all 51 FVE and WVA members, composed by national veterinary associations and several international organizations and corporations with a request to forward to their respective members, which resulted in additional responses from veterinary clinics, which included self-employed practitioners, practitioners working in corporate veterinary clinics (non-probability snowball sampling). Participants were given appropriate project information, including content, sponsorship, and purpose. Participation and each question were voluntary and not remunerated. Data were collected anonymously, unless participants wished to provide an email address of own accord and with informed consent. Any potential contact details or names mentioned by participants during the research were anonymized after transcription. A total of 10 questions in English covered demographics, the availability of support and importance of MWB and DEI, support implementation and design strategies, and impact of support programs on one single page (Supplementary Table S3). The impact was to be assessed based on the evaluation outcomes on institutional level with a numerical scale from 1 to 5 and reported as a median value for each category, where 1 implied no impact and 5 a very positive impact. Responses were editable by the participants until the survey was closed. Data were tabulated and visualized in Microsoft Excel. Incomplete or duplicate responses based on time stamps were removed and the first entry was kept for analysis. Responses were organized by submitting entities (veterinary organizations or veterinary clinics) and regions of origin (Asia, Africa, Europe, Middle East, North America, Oceania, and South America). The options of “yes” and “partially” were combined into a single category to create a binary system for data analysis for the question on importance of MWB and DEI.

### Interviews

Following survey completion, 14 targeted, virtual interviews were held with representatives of veterinary associations, companies, or global support programs in which three representatives each represented two organizations, resulting in feedback from 17 organizations (Figure 2). The interviewees were selected based on the online survey of all those who had support programs available on a voluntary basis. Using a structured approach, each participant was asked 3–5 questions (Supplementary Table S4), depending on the type of support program offered, with relevant follow-up questions asked when appropriate. Representatives rated their support program impact on a numerical scale from 1 to 5, where 1 implied no impact and 5 a very positive impact, based on institutional as well as personal evaluation, and reported on the current challenges and future goals of their organizations. Reports and quotes from the interviews were approved by the interviewees and collected to provide a broad overview of the MWB and DEI status in the represented countries and regions. Data from the interviews were analyzed using a thematic approach to identify recurrent themes from across participants.

### Webinars

One webinar each on MWB and DEI was organized by FVE in cooperation with Zoetis. The webinars gathered further information on the availability of veterinary support programs and their impact and allowed for an interactive session to exchange information between organizers of such programs. An interactive presentation tool (Mentimeter) was used to solicit anonymous and informal real-time feedback from the participants in both webinars. During the webinars, several programs related to MH and DEI were each presented by three subject matter experts and further discussed. A panel discussion each consisting of two members and audience participation took place to discuss selected statements and anonymized quotes were analyzed. Full programs and statements can be found in Supplementary Table S5.

## Results

### Survey

A total of 146 survey responses were received of which 137 responses met the inclusion criteria. Most responses (81%, *n* = 112/137) were received from veterinary organizations and veterinary clinics based in Africa and Europe (Table 1).

**Table 1:** Survey responses per region of the world and responding group.

**Region of the world**	**Veterinary organizations**	**Veterinary practitioners**
Africa	25 (32%)	36 (62%)
Asia	7 (9%)	0 (0%)
Europe	36 (45%)	15 (26%)
Middle East	0 (0%)	0 (0%)
North America	4 (5%)	0 (0%)
Oceania	6 (7%)	5 (9%)
South America	1 (1%)	2 (3%)
Total	79 (100%)	58 (100%)

#### Survey results on MWB

**Figure 3:**
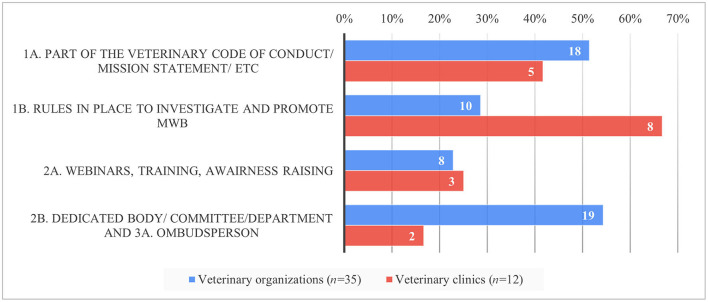
Relative proportion of MWB strategies implemented per tier and respondent group in per cent (*y*-axis) and absolute numbers (inside the bars) based on the survey responses.

More veterinary organizations (54%, *n* = 43/79) vs. veterinary clinics (26%, *n* = 15/57) explicitly designated a dedicated budget within their organization to MWB challenges including key priority areas such as professional stress, burnout, depression, compassion fatigue, and suicidal ideation (Q2a). More veterinary organizations (34%, *n* = 27/79) had already implemented committees, coaches, and education and training programs to increase the awareness of and improve MBW vs. veterinary clinics (12%, *n* = 7/57) (Q4a). A total of thirty-five (35) veterinary organizations (44%) and 12 veterinary clinics (21%) detailed the design of their support programs (Q2b). Most veterinary clinics implemented MWB activities by having rules in place to investigate and promote MWB (67%, *n* = 8/12) Whereas, veterinary organizations implemented this equally frequently as a part of their veterinary code of conduct, mission statement, etc. (51%, *n* = 18/35) and by having a dedicated body/committee/department or ombudsperson (54%, *n* = 19/35) (Figure 3). Both groups were least likely to use webinars, training, and awareness raising (activities of the 2nd tier).

**Figure 4:**
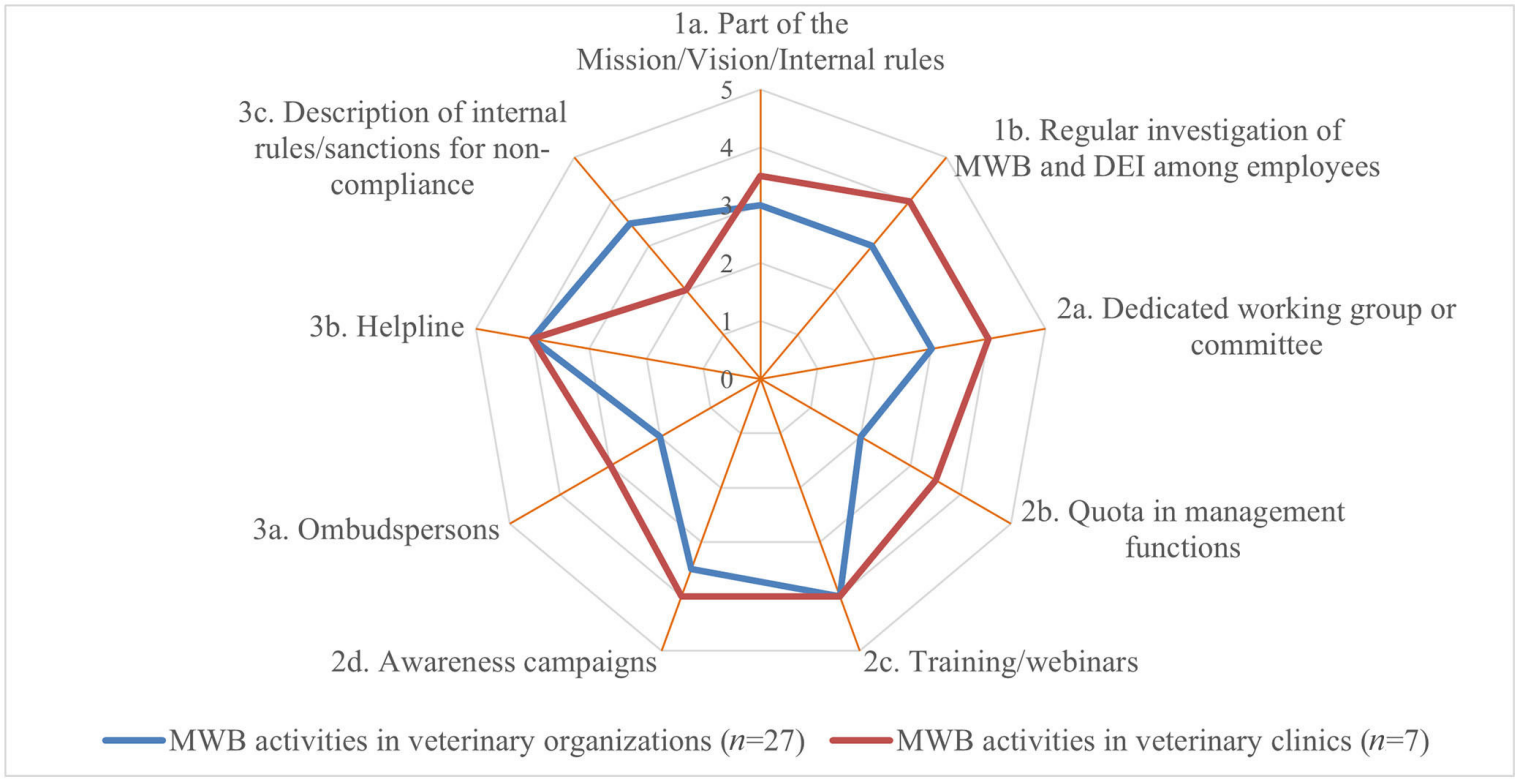
Perceived median impact of different MWB implementation strategies per tier and per respondent group (where 1 implied no impact and 5 a very positive impact) based on the survey responses.

Whereas, some activities of the 1st tier (such as stating MWB as part of the mission, vision or internal rules) were implemented frequently in both groups, regular investigation of MWB and non-discrimination of employees (e.g., through annual surveys) was less often implemented by veterinary organizations. However, both activities were scored high with a median impact of 3.5 and 4, respectively (Q6a). Activities of the 2nd tier were perceived as having a high impact (median of 3.5–4) by practitioners but were least implemented. Dedicated working groups or committees were perceived of lower impact with a median of each 3 but were implemented by the majority of veterinary organizations. The MWB impact of helplines for individuals (3rd tier) was scored highest with a median of 4 by both groups whereas, in sharp contrast to the veterinary organizations, ombudspersons were scored higher by veterinary clinics than description of internal rules/sanctions foreseen in cases of non-compliance for individual cases (Figure 4).

#### Survey results on DEI

**Figure 5:**
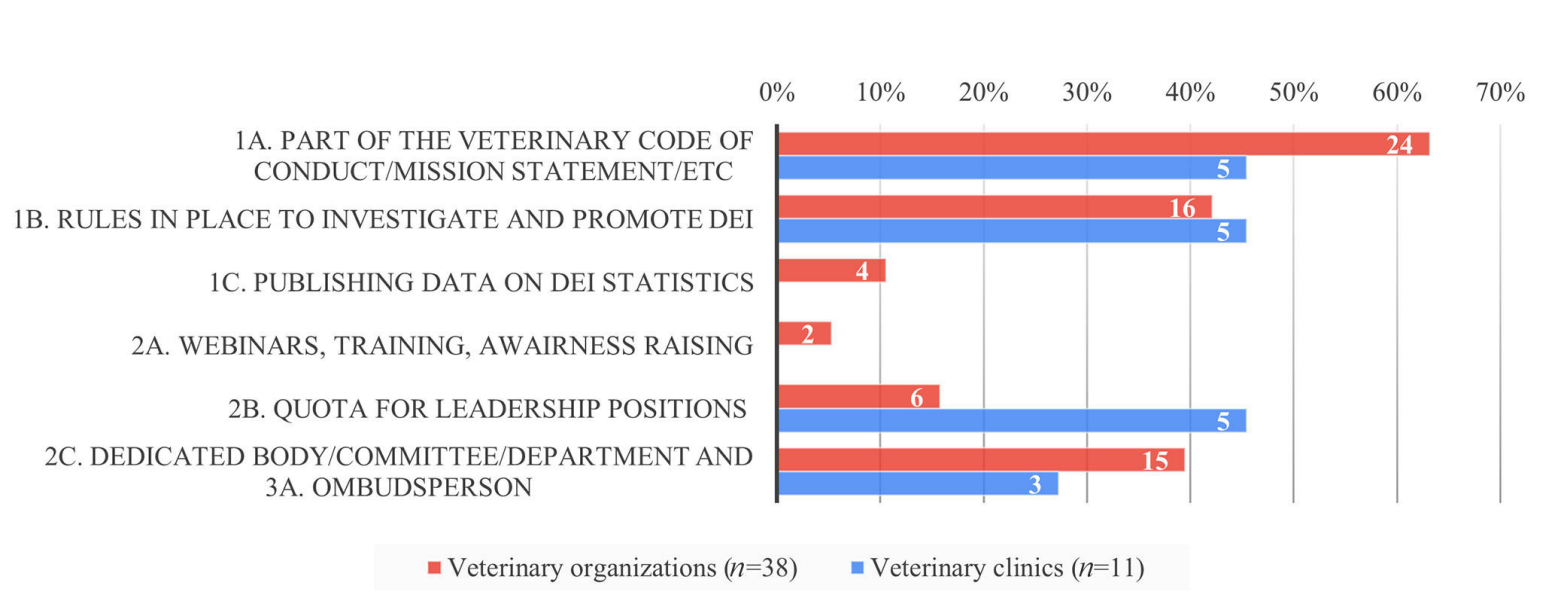
Relative proportion of DEI strategies implemented per tier and respondent group in per cent (*y*-axis) and absolute numbers (inside the bars) based on the survey responses.

More veterinary organizations (58%, *n* = 45/78) explicitly specified DEI (age, disability, race, ethnicity, gender, sexual identity, sexual preference, religion, personality, lifestyle, etc.) as a key priority area with a designated budget as opposed to veterinary clinics (33%, *n* = 19/57, Q3a). However, there was less difference between veterinary organizations (25%, *n* = 20/79) and veterinary clinics (14%, *n* = 7/58) who had already implemented committees/coaches/education and training to increase and improve DEI (Q5a). A total of thirty-eight (38) veterinary organizations (58%) and 11 veterinary clinics (19%) detailed the implementation design (Q3b). Most veterinary organizations implemented DEI activities as 1st tier activities such as being part of the veterinary code of conduct, mission statement, etc. (63%, *n* = 24/38) followed by having rules in place to investigate and promote DEI (42%, *n* = 16/38). In contrast, veterinary clinics implemented DEI equally frequently (45%, *n* = 5/11) by two 1st tier activities (1A and 1B) and one 2nd tier activity (2C). Similar to the MWB implementation, webinars, training, and awareness raising as activities of the 2nd tier were used least by both groups (Figure 5).

**Figure 6:**
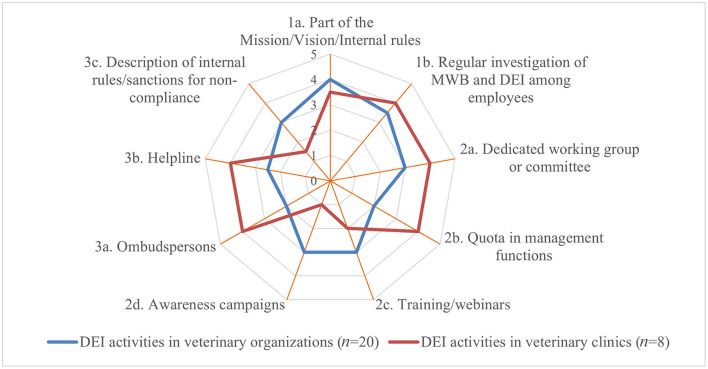
Perceived median impact of different DEI implementation strategies per tier and per respondent group (where 1 implied no impact and 5 a very positive impact) based on the survey responses.

The most frequently implemented 1st tier activities (Figures 1A,B, 5) were as well perceived as having the highest impact with a median of 4 and 3.5, respectively, by both groups (Q6b). Though seldomly implemented, 2nd tier activities such as awareness campaigns and training/webinars were perceived of high impact by veterinary clinics and of moderate impact by veterinary organizations. In contrast, veterinary clinics perceived the impact of 3rd tier activities such as individual counseling by ombudspersons and helplines higher with a median of each 4 than veterinary organizations, whereas the latter implemented these more frequently (Figure 6).

### Interview results

In the 14 semi-structured interviews, the representatives of 17 organizations who gave an overview on their MWB and DEI support programs, including eight national and one regional veterinary associations, two private veterinary MWB organizations, two veterinary pharmaceutical companies, two universities, one helpline, and one veterinary student association were interviewed (three representatives presented support programs for two organizations), in which specific MWB and DEI support programs were available. However, DEI support programs were less often implemented by these 17 veterinary organizations. Most representatives interviewed struggled to rate the impact of their support programs as only participation data were available, especially for DEI support programs (Table 2).

**Table 2:** Perceived impact of support programs by interviewed organizations per tier and implementation strategy.

**Target audience**	**Implementation strategy**	**Implementing organizations**	**Perceived Impact**	**Non-exhaustive alphabetic list of organizations and further info**
**3^**rd**^ tier - Individual services**	**Helpline**	MWB - 8/17	Effective to help individual veterinarians in need. No impact studies on how many veterinarians in need consulted the helpline or how effective the advice given by the helpline was.	AVMA, Beyond Blue with Zoetis Australia, Government of Costa Rica, Help4vets, HVA Greece, SAVA, Veterinary Ireland, Vetlife
	**Peer-to-peer support groups**	MWB - 5/17	Peer-to-peer support groups perceived as effective to help individual veterinarians in need.	AVMA, IVSA, KNMvD, SAVA, Veterinary Ireland
	**Legal support for individual veterinarians**	MWB – 1/17	Individual lawyer assistance considered as very helpful. Numbers of consults/year and success rate are not monitored consistently.	HVA Greece
**2^nd^ tier - Specific group services**	**Webinars**	MWB – 8/17 DEI – 3/17	Very effective in creating awareness and destigmatization, but less effective in solving or preventing MWB problems in individual veterinarians or DEI problems.	MWB: AVMA, Colegio de médicos veterinarios de Costa Rica, IVSA, KVA, MMI, SAVA, SVA, Zoetis DEI: AVMA, IVSA, SVA
	**Support communities**	DEI – 1/17	Specific communities to support DEI	Interviewees named several support communities (not always connected to their organization) such as Up&Out South-Africa, PrideVMC, DVMC, BVEDS, UK LGBTQI+, UCD LGBT Ireland
	**Dedicated MWB/DEI working group/ committee**	MWB - 6/17 DEI - 4/17	Effective to raise awareness and discuss how to tackle MWB problems, but no impact studies were available overall.	MWB: AVMA, FECAVA, IVSA, KVA, Utrecht University, SVA in cooperation with Norway and Denmark, DEI: AVMA, IVSA, KVA, Utrecht University
	**Specific support programs:** - Young graduates - Train the Trainer workshops - Veterinary mental health workshops	MWB - 3/17	Training program Ready – Vet – Go as a combination of practical information, mental coaching One-day interactive session to become educators in workplace communication skills that support wellbeing and learn how to use communication to help teams and individuals flourish and achieve their full potential Zoetis collaboration with key stakeholders leading to changes in curriculum for continuing education programs (to be started in 2021) and a national campaign to help the public gain a greater appreciation for the contributions and value of veterinarians.	Utrecht University in collaboration with KNMvD AVMA with MSD Zoetis
	- AVMA Workplace Well-being Certificate program - AVMA Brave Space Certificate program	MWB & DEI - 1/17	Programs focus on both MWB and DEI and comprised of multiple modules that can be taken individually or completed as a unit to improve MWB in the workplace. Popular: 2000+ courses taken since 2019. Impact not evaluated.	AVMA with MSD
**1^st^ tier: Services for all veterinary professionals**	**Regular investigation of MWB and DEI among employees**	MWB - 7/17 DEI – 3/17	Important to get information on the state of play regarding MWB of the profession.	MWB: AVMA with MSD, Colegio de médicos veterinaries de Costa Rica, IVSA, MMI, Utrecht University, VCI, Zoetis DEI: AVMA, IVSA, RCVS
	Dedicated wellness week	MWB - 2/17	Effective in creating awareness, unknown impact on solving MWB problems of individual veterinarians in need.	IVSA, Utrecht University
	**Specific support programs:** - Well-being Award - Awareness campaign “Respect for the Vet Profession”	MWB - 2/17	Awards a practice that has done extra efforts to ensure staff wellbeing. Foster the awareness of the respect for veterinarians and the veterinary profession and support their important contributions to animal health during unconventional times.	RCVS and the Society of Practicing Vets (SPVS) MSD
	- SafeVet Smart booklet - Online resources	MWB & DEI - 2/17	Handbook with easily applicable tools to improve MWB in veterinary practice. Considered impactful, but no concrete studies. Guide to manage work stress, and self-protection techniques	VCI Help4Vets
	**Specific support apps:** - MMI Kite App - VETVANCE App	MWB - 2/17	Go-to well-being tool which makes it possible to work on your specific needs. Popular (1K+ downloads), impact not measured Podcasts and courses on professional stewardship and personal wellness. Popular (1K+ downloads), but impact not measured	MMI VETVANCE
	- Research grants	MWB 3/17	Sponsorship of research focusing on developing necessary resources to improve veterinary MWB and/or support wellness programs and retreats at veterinary schools.	Zoetis, MSD, MMI

#### Webinar results

##### MWB webinar

The veterinary MWB webinar participants were most likely to list “low pay” (*n* = 12/42, 29%) and “long hours” (*n* = 10/42, 24%) as the most significant contributing factors to unfavorable MWB among veterinarians. Many of the participants stated that mental health support programs are available in their country (*n* = 13/14, 93%), but the attendance frequency was unknown (*n* = 7/13, 54%). During the panel discussion, four statements were discussed by two panel members with a contribution of the speakers, chair, and other participants (Table 3).

**Table 3:** Panel discussion outcomes of the MWB webinar.

**Statements**	**General outcomes**
*What have you seen that is working, in terms of supporting mental health and wellbeing amongst the veterinary team? Why do you think it is effective?*	- Improve our veterinary work culture - Destigmatization, improve help seeking behavior - Evaluation of programs and quantification of intervention impact
*How do we better communicate the skills required to be a successful veterinarian to potential vet students before starting and better support them through veterinary school?*	- Change of environment instead of increasing resilience - Veterinary specialists as role models - Undergraduate training in mental health literacy - Peer-to-peer groups to support young vet's MWB
*How can veterinary associations assist employers in their efforts to provide a mentally healthy workplace with the demands of clients and the reality of the job?*	- MWB support should be available and managed on national level - Focus on compulsory CPD, skills building, - Veterinary well-being awards to reward employers
*If we had to focus our efforts in one area, what do you think it should be?*	- Focus on the issues of staff well-being and encouraging others to replicate what their colleagues have started successfully

##### DEI webinar

The DEI webinar participants indicated that the most common associations made with DEI were “respect” (*n* = 20/57, 35%) and “belonging” (*n* = 18/57, 32%). The results showed that most participants (*n* = 15/19, 79%) stated that DEI was an important topic for the profession but an area for which more work is needed. During the panel discussion, three statements were discussed by two additional panel members with a contribution of the speakers, chair, and other participants (Table 4).

**Table 4:** Panel discussion outcomes of the DEI webinar.

**Statements**	**General outcomes**
*Is DEI an easy to talk about topic?*	- DEI is a cultural issue and it is challenging to get to the root cause - We do not know what we do not investigate
*Which support programs are most effective in improving DEI in the veterinary profession?*	- Dedicated lectures - Programs that create opportunities for conversations - AVMA Brave Space certificate program
*Do DEI support programs need to focus mainly on the leading generation or the future generation?*	- Students are the future profession, but they need examples from leaders in the profession to create role models and the future generation too. - In BVLGBT+ often the students on the committee drive the change.

## Discussion

### Availability and impact of support programs

Our study moved the focus from the identification of MH and DEI related issues in the veterinary profession to the action and intervention established through MWB and DEI support programs available for the three identified tiers (programs for the profession at large, for specific groups at risk, or for individual veterinarians). This was investigated on a national, regional, or global scale and explored information on the perceived impact of the existing programs.

The availability of studies on MWB support programs became more prominent in English-speaking countries over the last decade. In these countries, results from multiple studies and reviews detailing work-related stressors (3, 4, 6, 7, 10, 15, 16, 50–54), particularly during the COVID-19 pandemic (20–22, 24) emphasized MWB concerns within the veterinary profession. This was mirrored in our survey, with more responses on implemented strategies received from English-speaking countries. As our survey was solely distributed in English, this could have given a geographical disbalance. Research from larger veterinary organizations in English-speaking countries (11–13, 37, 55, 56) revealed increased psychological distress among veterinarians. This led to many organizations regardless their size establishing MWB support programs for veterinarians on an individual and/or workplace level, as well as the growth of large private organizations focusing on the MWB of veterinarians (MMI; Vetlife; and Not One More Vet). Though it is recognized that veterinary clinics face MWB challenges, the possibility to implement intervention measures is most likely hampered due to workforce shortages. However, larger veterinary corporate companies on a central level made major efforts in the recent years to tackle these issues (IVC Evidensia; AniCura).

Reports on the lack of DEI within the veterinary profession are as well more frequent from English-speaking countries, mostly coming from veterinary schools (26, 29, 57–63), but also from veterinary workplaces (27, 43, 64). Our study shows that in Europe, less organizations perform research on DEI and/or have support programs, though the results could have been biased due to the questionnaire being available solely in English. The identified DEI support programs were mostly targeting the inclusion of the LGBTQI+ community and different ethnicities (PrideVMC, DVMC, BVLGBT+; BVEDS, LGBT Ireland). No programs were identified tackling discrimination of other specific groups (e.g., veterinarians working in the city vs. rural areas, young vs. old, full-time vs. part-time, and specialists vs. generalists). In the EU, the Charter of Fundamental Rights lays down that any discrimination based on any ground shall be prohibited, obliging the EU MS to enact legislation including all grounds of discrimination envisaged in this fundamental right (65). This supranational Charter explains partly why there are less specific DEI support programs in the EU on national level. Legal obligations are present as well in other parts of the world but not always (fully) implied in practice due to different reasons, DEI support programs of the 1st tier were still in their infancy in some regions of the world, most likely as dialogs about gender, sexuality, and ethnicity are considered taboo. In addition, the cultural acceptability of extreme positive or negative opinions of assessment scales, cross-cultural equivalence of the categories “high” and “low” based on a scale from 1 to 5, and whether respondents could be biased toward answering questions in ways that are socially acceptable, particularly in the interviews, could have influenced the responses. One interviewee indicated:

“*In my country, DEI is still a taboo and difficult to talk about topic within the whole society, more specific the rural areas outside the big cities and among the older generations. Support programs are not (yet) available on DEI.”*

Whereas, DEI implementation strategies of all three tiers, including “part of the mission, vision or internal rules,” “dedicated working groups or committees” as well as “description of rules and regular investigation of non-discrimination of employees” were perceived as having a high impact, they were primarily implemented by veterinary organizations, possibly due to their complexity and time intensive maintenance.

Few studies (47–49) exist on the impact and effectiveness of different support programs for MWB and DEI, which was confirmed by our study. Program organizers found it challenging to measure the impact of their own programs as it is difficult to implement meaningful scoring metrics (what to measure: reduced suicide rates, improved veterinary resilience, perceived happiness?). Primarily, respondents could only evaluate the frequency with which their programs were used (e.g., how many participants certain webinars had, how many calls to a helpline, etc.).

First tier activities such as “description of internal rules/sanctions” were often implemented by veterinary organizations and were perceived as having a high impact. Veterinary clinics also frequently implemented them, but they perceived the impact of those internal rules to be much lower, probably due to the smaller work structure and more direct contact between the colleagues. The MMI Kite App is accessible for every veterinarian and a helpful tool to monitor MWB individually and guide to tailored assistance if needed. Tools intended for veterinary clinics such as the SafeVet Smartbooklet were also perceived as beneficial. For example, one participant indicated:

“*The SafeVet Smartbooklet spread among Irish veterinarians is considered very effective and impactful in creating awareness on mental health problems and providing tools easy applicable in practice. (Veterinary Council of Ireland)”*

With respect to second tier activities, tailored programs should be offered to higher-risk groups to deliver more targeted interventions. Webinars were identified as successful group programs with a very high potential impact (median of 4) to raise awareness and promote destigmatization. Surprisingly, this was the 2nd tier activity less frequently implemented by both veterinary organizations and clinics. Specific advantages of webinars are that they are cost-effective, can have a large outreach, and are universally available regardless of the geographical location of the veterinarian or speaker. More seasoned members of the profession can easily share their expertise on coping mechanisms with stressors and well-being optimization strategies. A before-after evaluation of outcomes and effects is imperative to monitor the developments over time (66). By way of support is the following quote from one student organization:

“*IVSA considers the Wellness Week as most successful program because it is attended by students from 15-20 countries. During this Week, there are 5 days of activities/online webinars with another theme daily. (IVSA)”*

The AVMA Workplace Well-being Certificate, a program to create a culture of well-being in the workplace, was also perceived as having a high impact. For example, one participant indicated:

“*For AVMA the Workplace Well-being Certificate program is the most popular and most attended program. The impact of creating awareness and starting the dialog to talk about mental health issues is large. The majority of feedback received indicates the program as ‘very helpful' to participants. AVMA is now moving away from the individual vet focus towards a focus on the whole veterinary team and practice. (AVMA)”*

Different studies report **peer-to-peer** coaching as being effective in tackling mental health problems in students (36, 67, 68), in general practitioners, especially in teaming up with mental health support research (69, 70) and for other healthcare professionals (71–74). Training students in specific group session format were identified as a useful teaching method especially when concrete skills were emphasized, such as self-care and well-being (68, 75). Platt et al. interviewed 21 British veterinarians with mental health problems about coping mechanisms, and they indicated that peer support was beneficial (76). In a recent study, 889 US veterinarians indicated that they would cope with a distressing situation by talking with a partner or friend (72% of respondents), and/or by discussing with a colleague (9). In the US, it was recommended that veterinary practitioners should connect with others to minimize the development of compassion fatigue, and this should include debriefing after critical incidents and having supportive conversations at team meetings (77). The potential impact of peer-to-peer exchange was highlighted in a recent study (78), in which it was found that final year veterinary students improved their communication skills significantly after peer-to-peer learning with role-play simulations with other students about breaking bad news. However, students commonly choose to speak to a peer or a family member for MWB issues instead of a health professional because of concerns over perceived lack of confidentiality, fears of documentation of their MWB status, or concerns that disclosure will affect their academic prowess or their career (79, 80). This perceived stigma, whether real or imagined, hampers help-seeking behavior.

**Figure 7:**
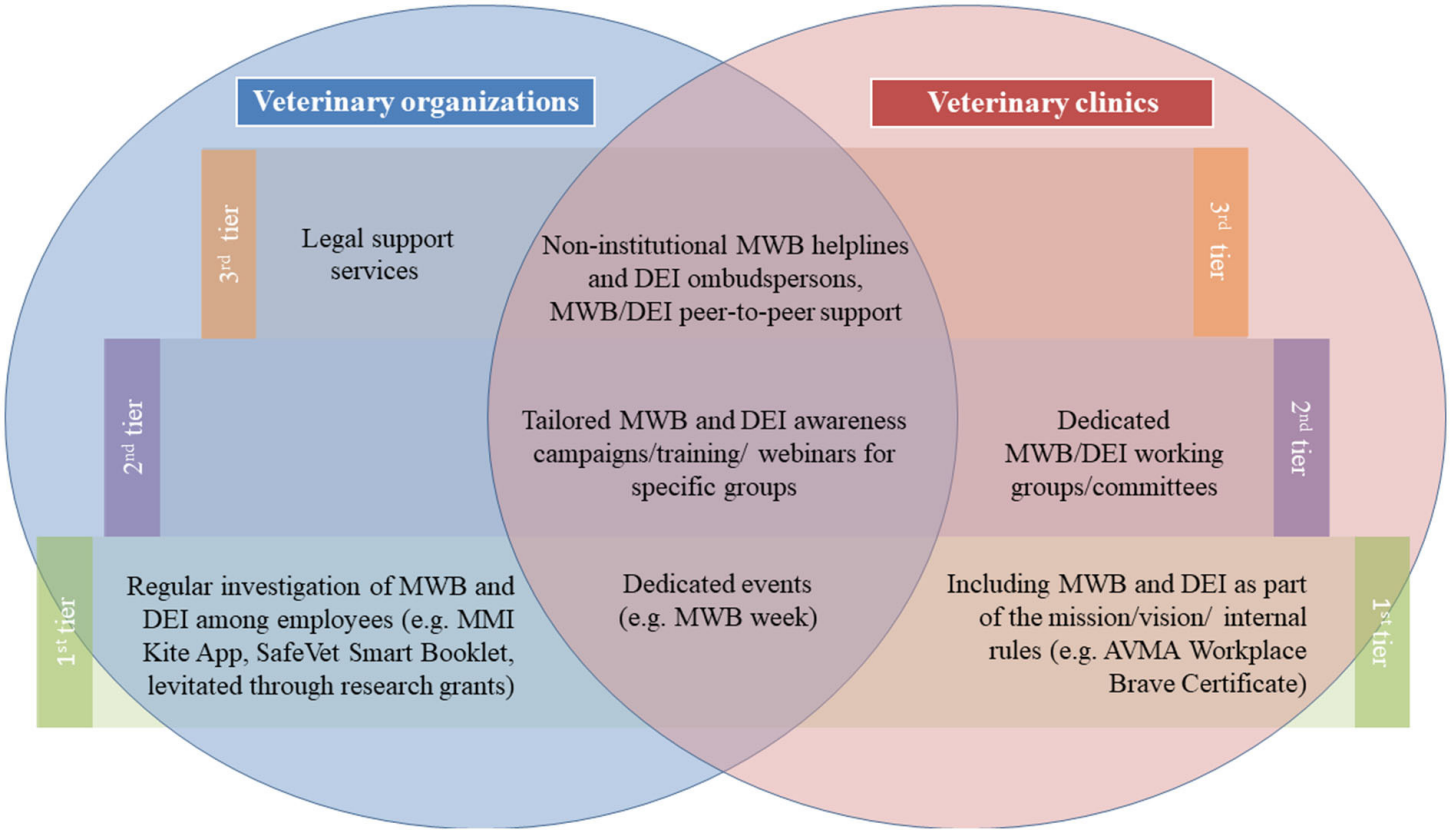
Venn diagram of MWB and DEI support programs that were identified to be most importantly implemented per tier and per group based on the survey, interview, and webinar outcomes.

Third tier activities such as helplines were perceived as having the highest impact for DEI and MWB. However, individual support programs were infrequently implemented, probably either due to the cost involved to train, staff, and maintain the service or the existence of alternatives (e.g., general suicide helplines) (Figure 7).

### Focusing on the formative years of the veterinary career to enable a resilient, future-proof, and thriving profession

In our first webinar, the most important factors related to unfavorable MWB were “low pay,” “long hours.” Work-related factors such as “long hours,” “heavy workload,” and “imbalance work-life/private life” have been previously reported to contribute to unfavorable MWB (4, 50, 81–84). Therefore, programs should be provided for all veterinarians beginning with undergraduate training, reinforced at annual conferences, and/or by offering continuing professional development on the subject for veterinary clinics and practitioners. There is good reason to focus on the formative years of the veterinary career, as MWB interventions in this period seem to lead to the longest beneficial effects and are the easiest to implement (85). It is an imperative for professional organizations as well as for veterinary clinics to set and defend minimum standards for employments and encourage early career professionals to value themselves and vouch for fair working conditions. However, veterinary students should be prepared for challenging situations that come inevitably with their future work and embrace lifelong learning, including soft skills. Undergraduate courses on MWB including topics on coping styles, early identification of signs of stress in self and others, and enhancing help-seeking behaviors were suggested for all students as a part of the core curriculum (7). Resilience training has been stated as successful (86) and is considered as a part of selection procedures for students of many health programs (87). While avoiding discrimination of applicants based on their psychological health profile, resilience is considered to be an imperative for veterinary studies (88). The next generation of veterinarians grows up in a society with certain expectations about values, team spirit, a work schedule that also allows time spent with family and friends, fair remuneration, and possibilities to change to other veterinary work fields or professions. This demands a culture change within veterinary medicine and veterinary practice [also earlier stated by (15, 89)], in which a compensation model that incentivizes long hours and promotes heroism should not be used. Several authors (36, 48, 90) have reported on the “Job Demands Resources Model” (91) and its successful use in “Job crafting” within (early career) veterinary professionals, which was reported as a promising and positive intervention within other professions (92).

The second webinar revealed that factors related to DEI were positively connotated (“respect” and “belonging”). Strengthened networks between underrepresented minorities within the veterinary profession could be alleviated by mentor–mentee programs, in which fostering a sense of belonging can improve well-being (93). The support programs should focus both on the current generation and on the future generations as it is important to create role models within the profession, as well as recruiting a more diverse pool of veterinary students who will later represent the veterinary profession. An explicit offer of 2nd tier resources and strategies to implement pipeline programs increasing diversity according to evidence-based practices will foster a welcoming and inclusive environment. Third tier strategies such as individual consequences for not adhering to expected policies are imperative to establish this standard. In other (medical) professions, different support programs on all tiers were used and stated as effective. This includes recruiting a large group of minorities (94, 95), creating a safe and antiracist work environment (96), training staff to recognize unconscious bias and exclude discriminatory behavior (97–99), and using DEI to create workplace targets as well as embracing it as a part of organizational strategy (100). Improving diversity starts with the selection and admission to veterinary schools to change gradually the demographics of the profession. This 1st tier implementation strategies are also used and recommended in other medical professions (101). Willbur et al. (102) stated that increasing the diversity within the healthcare workforces would require social, academic, and financial support, which was recommended earlier by Elmor et al. (27) to improve diversity within the US veterinary schools and profession. This is supported by Witte et al. (64) who concluded that LGBTQI+ support programs were more available within the US universities compared to the professional environment. The improvement of diversity must tackle this inclusion of a wide group of minorities, including the LGBTQI+ community but also underrepresented ethnicities, people with disabilities, people originating from less favorable socioeconomic backgrounds, etc.

### Recommendations

In general, interventions should be undertaken by veterinarians as individuals and by veterinary organizations to be most effective as this will best incorporate interventions that address all three strategic tiers (103).

1: *Veterinary organizations should continue raising awareness and alleviate stigma associated with mental well-being discussions through webinars, focused training, and broad continuing education at national/regional level*

Webinars and group trainings are very useful tools to raise awareness of MWB and DEI and for reducing stigma, which is important to create a culture of sharing, belonging, and talking. In addition, when delivered as dedicated Continuing Professional Development (CPD) courses providing credit points, the approaches and techniques can incentivize and help veterinarians throughout their professional life to develop coping styles, early identification of signs of stress in themselves and others and enhance the likelihood of help-seeking behaviors.

2: *Veterinary practitioners need more practical, tailored tools, and resources*

More 2nd tier activities such as tailor-made awareness campaigns for specific groups at risk, peer-to-peer groups (ideally with dedicated professional support, e.g., psychologists), but as well the promotion of 1st tier activities such mobile application and initiative such as the SafeVet Smartbooklet, are recommended to specifically address the needs of the veterinary profession and provide MWB and DEI support. These should be practical and tailored to the needs of the individual and easy to find and use in practice.

3: *Steps toward improved MWB and DEI are a necessity for a thriving, future-proof profession*

As a profession, MWB should be prioritized during initial veterinary training and throughout the professional career. In addition, veterinary organizations, and, in particular, veterinary faculties, should work collaboratively with practitioners to exchange, support, and ensure that evidence-based methods are implemented to increase DEI in the veterinary profession. DEI support programs should focus on incentivizing 1st tier activities targeting the current generation as well as the future generation.

### Limitations of this study

The non-probability snow-ball sampling of the survey made it difficult to determine the sampling error or generalize inferences about the studied entities based solely on the questionnaire responses obtained. The overall picture of MWB and DEI support programs presented in the survey and in the additional in-depth interviews was warranted, although we acknowledge that the responses may reflect the views of those veterinary organizations and veterinary clinics that are more proactive in promoting MWB and DEI. In the survey as well as in the interviews, there was a subjective measurement bias in the results since respondents were asked to rate the impact of their own support programs. In fairness, it is very difficult to quantify the impact of such support programs, especially over the short term. The impact may only be visible by repeating surveys and viewing the results after several years have passed to evaluate changing trends. The accessibility and geographical coverage of the survey could possibly be improved by providing the questionnaires in different languages. However, even well-translated surveys can be biased by cultural issues. The main considerations were the acceptability of extreme positive or negative opinions of assessment scales in various cultures, cross-cultural equivalence, and whether respondents could be biased toward answering questions in ways that are socially acceptable in the interviews. By design, the interviews did not allow for quantifiable differences between participants, but they provided the richness and depth necessary to inform and motivate future studies to explore the impact of MWB and DEI disparities.

## Conclusions

Openness to MWB and DEI issues is prerequisites for a striving profession. Attention to veterinary MWB and DEI has increased substantially in the last decade, followed by the development of support programs by many veterinary organizations, companies, and faculties. Results from the English-speaking countries spotlighted the importance of MWB and DEI in the veterinary profession, but more research is needed in many other countries, especially those in which some aspects may still be taboo. Implementation strategies to increase awareness of MWB and DEI must reach all veterinarians at all levels of their professional careers. Positive rewarding programs help to raise awareness, e.g., rewarding “good workplaces” with employers or organizations that “go the extra mile” to create an inclusive and positive workplace for their team. In particular, 2nd tier activities such as webinars were universally stated as being very effective in creating awareness and having a large impact. Individual veterinarians in need will highly benefit from a tailored approach (e.g., helplines, peer-to-peer support groups). DEI support programs are currently less available to create opportunities for open conversations. At the beginning of veterinary training, all students need positive and inclusive role models as well as diverse examples from the veterinary profession. More research is needed to design an objective universal applicable scoring system to objectively rate the impact of different support programs available and elaborate evidence-based statements. MWB and DEI should be seen in a wider perspective, and a change will also be needed in the veterinary work culture and environment, so that veterinary professionals can thrive in the profession.

## Data availability statement

The datasets analyzed for this study are available on request from the corresponding author, NDB.

## Ethics statement

The Ethics Committee of the Cliniques Universitaires Saint-Luc (Brussels, Belgium) and the CHU UCL Namur (Yvoir, Belgium) confirmed that this non-interventional study was legally exempted from ethical review as laid down in Art. 10 of the Belgian law relating to experiments on the human person from 7 May 2004.

## Author contributions

Study conception and design: NDB and PT. Data collection, analysis, and interpretation of results: FT, WJ, and NDB. Draft manuscript preparation: FT, WJ, PT, and NDB. All authors reviewed the results and approved the final version.

## Funding

This research received partial funding from Zoetis, which was used for article processing costs, study recruitment, and workshop-related announcements and communications. The FVE had full editorial control over the study design, content, and analyses.

## Conflict of interest

Authors WJ and NDB were employed by Federation of Veterinarians of Europe. The remaining authors declare that the research was conducted in the absence of any commercial or financial relationships that could be construed as a potential conflict of interest.

## Publisher's note

All claims expressed in this article are solely those of the authors and do not necessarily represent those of their affiliated organizations, or those of the publisher, the editors and the reviewers. Any product that may be evaluated in this article, or claim that may be made by its manufacturer, is not guaranteed or endorsed by the publisher.

## Supplementary material

Supplementary Table S1 STROBE Statement—Checklist of items that should be included in reports of cross-sectional studies.

Supplementary Table S2 Checklist for Reporting Results of Internet E-Surveys (CHERRIES).

Supplementary Table S3 FVE/WVA/Zoetis survey questionnaire as agreed on the availability of MWB and DEI support programs and their impact.

Supplementary Table S4 Interviewed organizations and questions for interviewees who were selected based on the online survey of those who had support programs available.

Supplementary Table S5 FVE/Zoetis webinar programs and panel discussion statements.

## References

[1] Galli N. Foundations of health education. J Sch Health. (1976) 46:158–65. 10.1111/j.1746-1561.1976.tb02003.x 1044938

[2] Federation of Veterinarians of Europe. FVE VETSURVEY 2018. Brussels, Belgium: FVE (2019). Available online at: https://fve.org/cms/wp-content/uploads/FVE_Survey_2018_WEB.pdf (accessed February 21, 2022).

[3] Gardner DH Hini D. Work-related stress in the veterinary profession in New Zealand. N Z Vet J. (2006) 54:119–24. 10.1080/00480169.2006.36623 16751842

[4] Kersebohm JC Lorenz T Becher A Doherr MG. Factors related to work and life satisfaction of veterinary practitioners in Germany. Vet Rec Open. (2017) 4:e000229. 10.1136/vetreco-2017-000229 29018534 PMC5623335

[5] Nett RJ Witte TK Holzbauer SM Elchos BL Campagnolo ER Musgrave KJ. Risk factors for suicide, attitudes toward mental illness, and practice-related stressors among US veterinarians. J Am Vet Med Assoc. (2015) 247:945–55. 10.2460/javma.247.8.945 26421408

[6] Vande Griek OH Clark MA Witte TK Nett RJ Moeller AN Stabler ME. Development of a taxonomy of practice-related stressors experienced by veterinarians in the United States. J Am Vet Med Assoc. (2018) 252:227–33. 10.2460/javma.252.2.227 29319445 PMC5985521

[7] Bartram DJ Yadegarfar G Baldwin DS. Psychosocial working conditions and work-related stressors among UK veterinary surgeons. Occup Med (Lond). (2009) 59:334–41. 10.1093/occmed/kqp072 19482885

[8] Emmett L Aden J Bunina A Klaps A Stetina BU. Feminization and stress in the veterinary profession: a systematic diagnostic approach and associated management. Behav Sci (Basel). (2019) 9:E114. 10.3390/bs9110114 31739637 PMC6912712

[9] Moses L Malowney MJ Wesley Boyd J. Ethical conflict and moral distress in veterinary practice: a survey of North American veterinarians. J Vet Intern Med. (2018) 32:2115–22. 10.1111/jvim.15315 30320478 PMC6271308

[10] Perret JL Best CO Coe JB Greer AL Khosa DK Jones-Bitton A. The complex relationship between veterinarian mental health and client satisfaction. Front Vet Sci. (2020) 7:92. 10.3389/fvets.2020.00092 32158771 PMC7052013

[11] Robinson D Edwards M Mason B Cockett J Arnill Graham K Martin A. RCVS 2019 Survey of the Veterinary Profession. UK: RCVS (2019). Available online at: https://www.rcvs.org.uk/news-and-views/publications/the-2019-survey-of-the-veterinary-profession/ (accessed February 21, 2022).

[12] Volk J Schimmack U Strand E Siren C Lord L. Merck animal health veterinary wellbeing study I. J Am Vet Med Assoc. (2019) 252:1231–8. 10.2460/javma.252.10.1231 29701527

[13] Volk J Schimmack U Strand E Vasconcelos J Siren C Lord L. Merck Animal Health Veterinary Wellbeing Study II. Merck Animal Health USA. (2020). Available online at: https://www.merck-animal-health-usa.com/about-us/veterinary-wellbeing-study (accessed December 21, 2021).

[14] Best CO Perret JL Hewson J Khosa DK Conlon PD Jones-Bitton A. Survey of veterinarian mental health and resilience in Ontario, Canada. Can Vet J. (2020) 61:166–72. 32020936 PMC6973204

[15] Perret JL Best CO Coe JB Greer AL Khosa DK Jones-Bitton A. Prevalence of mental health outcomes among Canadian veterinarians. J Am Vet Med Assoc. (2020) 256:365–75. 10.2460/javma.256.3.365 31961276

[16] Hatch PH Winefield HR Christie BA Lievaart JJ. Workplace stress, mental health, and burnout of veterinarians in Australia. Aust Vet J. (2011) 89:460–8. 10.1111/j.1751-0813.2011.00833.x 22008127

[17] Dalum HS Tyssen R Hem E. Prevalence and individual and work-related factors associated with suicidal thoughts and behaviours among veterinarians in Norway: a cross-sectional, nationwide survey-based study (the NORVET study). BMJ Open. (2022) 12:e055827. 10.1136/bmjopen-2021-055827 34980627 PMC8724721

[18] Cevizci S Babaoglu ÜT Serpen A Yilmaz O Boyar H Çelikel S. Occupational stress and risk factors in veterinary surgeons. Kafkas Univ Vet Fak Derg. (2014) 20:41–8. 10.9775/kvfd.2013.9426

[19] Begeny C Ryan M Bongiorno R. Motivation, satisfaction and retention: Understanding the importance of vets' day-to-day work experiences. Washington, D.C., USA: BVA (2018). Available online at: https://www.bva.co.uk/media/2990/motivation-satisfaction-and-retention-bva-workforce-report-nov-2018-1.pdf (accessed February 21, 2022).

[20] Mair TS Mountford DR Radley R Lockett E Parkin TD. Mental wellbeing of equine veterinary surgeons, veterinary nurses and veterinary students during the COVID-19 pandemic. Equine Vet Educ. (2021) 33:15–23. 10.1111/eve.13399

[21] Mair TS Lockett E. The impact of COVID-19 on equine veterinary practice and mental wellbeing. Equine Vet Educ. (2021) 33:6–9. 10.1111/eve.13416

[22] Mureşan AN Morariu S Baisan RA Costea R Mureşan C. The impact of COVID-19 pandemic during lockdown on the veterinary profession in Romania: a questionnaire-based survey. Front Vet Sci. (2021) 8:737914. 10.3389/fvets.2021.737914 34859084 PMC8631325

[23] Quain A Mullan S McGreevy PD Ward MP. Frequency, stressfulness and type of ethically challenging situations encountered by veterinary team members during the COVID-19 pandemic. Front Vet Sci. (2021) 8:647108. 10.3389/fvets.2021.647108 33912607 PMC8071942

[24] Quain A Mullan S Ward MP. Risk factors associated with increased ethically challenging situations encountered by veterinary team members during the COVID-19 pandemic. Front Vet Sci. (2021) 8: 10.3389/fvets.2021.752388 34760959 PMC8573112

[25] British Veterinary Association,. BVA Report on Discrimination in the Veterinary Profession 2019. (2019). Available online at: https://www.bva.co.uk/media/2991/bva-report-on-discrimination-in-the-veterinary-profession.pdf (accessed February 21, 2022).

[26] Elmore RG. The lack of racial diversity in veterinary medicine. J Am Vet Med Assoc. (2003) 222:24–6. 10.2460/javma.2003.222.24 12523474

[27] Elmore RG. Reasons for the lack of racial diversity in veterinary medicine. J Vet Med Educ. (2004) 31:414–6. 10.3138/jvme.31.4.414 15551241

[28] Greenhill LM. DiVersity Matters: a review of the diversity initiative of the Association of American Veterinary Medical Colleges. J Vet Med Educ. (2009) 36:359–62. 10.3138/jvme.36.4.359 20054071

[29] Greenhill LM Davis KC Lowrie PM Amass SF (Eds,.). Navigating Diversity Inclusion in Veterinary Medicine. https://www.google.com/search?client=firefox-b-d&q=West$+$Lafayette&stick=H4sIAAAAAAAAAOPgE-LSz9U3MCootEhJUuIAsYsLUwy0tLKTrfTzi9IT8zKrEksy8_NQOFYZqYkphaWJRSWpRcWLWPnCU4tLFHwS0xIrU0tKUnewMu5iZ-JgAABPy3ADXQAAAA&sa=X&ved=2ahUKEwjXnqvPg_D4AhVmD1kFHfgjBhAQmxMoAHoECEcQA West Lafayette, IN: Purdue University Press (2013). 1 p.

[30] Snyder CR Frogner BK Skillman SM. Facilitating racial and ethnic diversity in the health workforce. J Allied Health. (2018) 47:58–65. 29504021

[31] German Veterinary Statutory Body (BTK). Statistik 2020: Tierärzteschaft in der Bundesrepublik Deutschland. Deut Tierärzteblatt. (2020) 5:558–568.

[32] Gül RTB Ozkul T Akçay A Ozen A. Historical profile of gender in Turkish veterinary education. J Vet Med Educ. (2008) 35:305–9. 10.3138/jvme.35.2.305 18723820

[33] Heath TJ. Longitudinal study of veterinary students and veterinarians: family and gender issues after 20 years. Aust Vet J. (2007) 85:290–5. 10.1111/j.1751-0813.2007.00180.x 17615043

[34] Irvine L Vermilya JR. Gender work in a feminized profession: the case of veterinary medicine. Gend Soc. (2010) 24:56–82. 10.1177/0891243209355978

[35] Lofstedt J. Gender and veterinary medicine. Can Vet J. (2003) 44:533–5. 12892284 PMC340187

[36] Mastenbroek NJJM. The art of staying engaged: the role of personal resources in the mental well-being of young veterinary professionals. J Vet Med Educ. (2017) 44:84–94. 10.3138/jvme.0216-041R1 28206838

[37] Robinson D Buzzeo J. Survey of Recent Graduates. UK: RCVS (2013). Available online at: http://www.rcvs.org.uk/publications/rcvs-survey-of-recent-graduates-ies-2013/rcvs-survey-of-recent-graduates-2013.pdf (accessed February 21, 2022).

[38] Veterinärmedizinischen Universität Wien,. Vetmeduni Vienna Jahresbericht 2020. Vienna (2020). Available online at: https://www.vetmeduni.ac.at/fileadmin/v/publicrelations/oeuk/Jahresbericht/Vetmeduni_Vienna_Jahresbericht_2020_WEB_low.pdf (accessed February 21, 2022).

[39] Williams A. Sexism straight from the horse's mouth: life as a female vet. The Conversation. (2014). Available online at: http://theconversation.com/sexism-straight-from-the-horses-mouth-life-as-a-female-vet-33962 (accessed December 21, 2021).

[40] Gatrell C. Embodying Women'S Work. UK: McGraw-Hill Education (2008). 225 p.

[41] Knights D Clarke C. Gendered practices in veterinary organisations. Vet Rec. (2019) 185:407. 10.1136/vr.104994 31501352

[42] Castro SM Armitage-Chan E. Career aspiration in UK veterinary students: the influences of gender, self-esteem and year of study. Vet Rec. (2016) 179:408. 10.1136/vr.103812 27516440

[43] Witte TK Kramper S Carmichael KP Chaddock M Gorczyca K. A survey of negative mental health outcomes, workplace and school climate, and identity disclosure for lesbian, gay, bisexual, transgender, queer, questioning, and asexual veterinary professionals and students in the United States and United Kingdom. J Am Vet Med Assoc. (2020) 257:417–31. 10.2460/javma.257.4.417 32715886

[44] Shergill Kaur N Buren L Martina M Sekinat Oluboyede I Saidi H. Report of the International Veterinary Students' Association's (IVSA) Taskforce on Diversity, Equity Inclusivity (DEI) - Discrimination in the veterinary community. (2019). Available online at: https://www.ivsa.org/wp-content/uploads/2021/09/IVSA-Discrimination-In-The-Veterinary-Community-f-1.pdf (accessed February 21, 2011).

[45] Moir FM Van den Brink A. Current insights in veterinarians' psychological wellbeing. N Z Vet J. (2020) 68:3–12. 10.1080/00480169.2019.1669504 31607215

[46] Kassem AM Witte TK Nett RJ Carter KK. Characteristics associated with negative attitudes toward mental illness among US veterinarians. J Am Vet Med Assoc. (2019) 254:979–85. 10.2460/javma.254.8.979 30938608 PMC6554711

[47] Halliwell REW Downes M Adams VJ Allister R Harrison W Mellanby RJ. Stress in new graduates: can the profession do more to help? Vet Rec. (2016) 178:635–6. 10.1136/vr.i3032 27313254

[48] Mastenbroek NJJM van Beukelen P Demerouti E Scherpbier AJJA Jaarsma ADC. Effects of a 1 year development programme for recently graduated veterinary professionals on personal and job resources: a combined quantitative and qualitative approach. BMC Vet Res. (2015) 11:311. 10.1186/s12917-015-0627-y 26717891 PMC4697329

[49] McKenzie A Allister R Humphrey D Moore K Greenberg K Greenberg N. An evaluation of a veterinary-specific mental health service. Occup Med (Lond). (2020) 70:169–75. 10.1093/occmed/kqaa017 32047935

[50] Arbe Montoya AI Hazel SJ Matthew SM McArthur ML. Why do veterinarians leave clinical practice? A qualitative study using thematic analysis. Vet Rec. (2021) 188:e2. 10.1002/vetr.2 34651756

[51] Chigerwe M Barter L Dechant JE Dear JD Boudreaux KA. A preliminary study on assessment of wellbeing among veterinary medical house officers. PLoS ONE. (2021) 16:e0253111. 10.1371/journal.pone.0253111 34166405 PMC8224950

[52] Dow MQ Chur-Hansen A Hamood W Edwards S. Impact of dealing with bereaved clients on the psychological wellbeing of veterinarians. Aust Vet J. (2019) 97:382–9. 10.1111/avj.12842 31364771

[53] Fritschi L Morrison D Shirangi A Day L. Psychological well-being of Australian veterinarians. Aust Vet J. (2009) 87:76–81. 10.1111/j.1751-0813.2009.00391.x 19245615

[54] Mastenbroek NJJM Jaarsma ADC Demerouti E Muijtjens AMM Scherpbier AJJA van Beukelen P. Burnout and engagement, and its predictors in young veterinary professionals: the influence of gender. Vet Rec. (2014) 174:144. 10.1136/vr.101762 24306199

[55] Dwyer A. AVMA AAEP Economics Report: Data on Member Wellness Parenting. Denver, CO (2019). p. 73–80. Available online at: https://aaep.org/sites/default/files/Documents/AAEP_Webinar_05-27-20_%202019_FINAL_AMVA_AAEP_Equine_Report.pdf (accessed February 21, 2022)/

[56] Robinson D Hooker H. RCVS 2006 Survey of the Professions. (2006). Available online at: https://www.rcvs.org.uk/news-and-views/publications/rcvs-survey-of-the-professions-2006/ (accessed December 22, 2021).

[57] Adams EW. A historical overview of African American veterinarians in the United States, 1889- 2000. J Vet Med Educ. (2004) 31:409–13. 10.3138/jvme.31.4.409 15551240

[58] Brown SE. Ethnic variations in pet attachment among students at an American school of veterinary medicine. Soc Anim. (2002) 10:249–66. 10.1163/156853002320770065

[59] Brown S-E. The under-representation of African Americans in animal welfare fields in the United States. Anthrozoös. (2005) 18:98–121. 10.2752/089279305785594225

[60] Greenhill LM Nelson PD Elmore RG. Racial, cultural, and ethnic diversity within US veterinary colleges. J Vet Med Educ. (2007) 34:74–8. 10.3138/jvme.34.2.74 17446630

[61] Greenhill LM Carmichael KP. Survey of college climates at all 28 US colleges and schools of veterinary medicine: preliminary findings. J Vet Med Educ. (2014) 41:111–21. 10.3138/jvme.0513-075R1 24855030

[62] Kendall T. Diversity and changing demographics: how they will affect veterinary medicine. J Vet Med Educ. (2004) 31:406–8. 10.3138/jvme.31.4.406 15551239

[63] Morse EM,. Minority Student Perceptions of the Veterinary Profession: Factors Influencing Choices of Health Careers. (2008). Available online at: https://www.semanticscholar.org/paper/Minority-Student-Perceptions-of-the-Veterinary-of-Morse/ec2faccbe851339a25b233f76635c63f60da62dc (accessed December 22, 2021).

[64] Witte TK Gorczyca K Chaddock M Greenhill LM Carmichael P. Health and Well-Being Among LGBTQ Veterinary Professionals: What it is and why it is Different. Denver, CO (2018). p. 4

[65] Bercusson B Blanke T Bruun N. European labour law and the EU Charter of Fundamental Rights. Baden-Baden, Germany: ETUI, The European Trade Union Institute, Nomos Baden-Baden (2006).

[66] Stigler Granados P Pacheco GJ Núñez Patlán E Betancourt J Fulton L. Assessing the effectiveness of Chagas disease education for healthcare providers in the United States. BMC Infect Dis. (2020) 20:743. 10.1186/s12879-020-05474-w 33036559 PMC7547496

[67] Schaub-de Jong MA Cohen-Schotanus J Dekker H Verkerk M. The role of peer meetings for professional development in health science education: a qualitative analysis of reflective essays. Adv Health Sci Educ Theory Pract. (2009) 14:503–13. 10.1007/s10459-008-9133-3 18766452 PMC2744783

[68] Siqueira Drake A Hafen M Rush BR. Promoting well-being among veterinary medical students: protocol and preliminary findings. J Vet Med Educ. (2014) 41:294–300. 10.3138/jvme.0214-026R 25000881

[69] Gillard S Holley J. Peer workers in mental health services: literature overview. Adv Psychiatr Treat. (2014) 20:286–92. 10.1192/apt.bp.113.011940

[70] Repper J Carter T. A review of the literature on peer support in mental health services. J Ment Health. (2011) 20:392–411. 10.3109/09638237.2011.583947 21770786

[71] Hu Y-Y Fix ML Hevelone ND Lipsitz SR Greenberg CC Weissman JS. Physicians' needs in coping with emotional stressors: the case for peer support. Arch Surg. (2012) 147:212–7. 10.1001/archsurg.2011.312 22106247 PMC3309062

[72] Scott SD Hirschinger LE Cox KR McCoig M Hahn-Cover K Epperly KM. Caring for our own: deploying a systemwide second victim rapid response team. Jt Comm J Qual Patient Saf. (2010) 36:233–40. 10.1016/S1553-7250(10)36038-7 20480757

[73] van Buschbach S van der Meer CAI Dijkman L Olff M Bakker A. Web-based peer support education program for health care professionals. Jt Comm J Qual Patient Saf. (2020) 46:227–31. 10.1016/j.jcjq.2019.12.005 32008958

[74] West CP Dyrbye LN Rabatin JT Call TG Davidson JH Multari A. Intervention to promote physician well-being, job satisfaction, and professionalism: a randomized clinical trial. JAMA Intern Med. (2014) 174:527–33. 10.1001/jamainternmed.2013.14387 24515493

[75] Liu AR van Gelderen IF. A systematic review of mental health-improving interventions in veterinary students. J Vet Med Educ. (2020) 47:745–58. 10.3138/jvme.2018-0012 32027214

[76] Platt B Hawton K Simkin S Dean R Mellanby R. Suicidality in the veterinary profession: Interview study of veterinarians with a history of suicidal ideation or behavior. Crisis. (2012) 33:280–9. 10.1027/0227-5910/a000143 22713972

[77] Kahler SC. Moral stress the top trigger in veterinarians' compassion fatigue: veterinary social worker suggests redefining veterinarians' ethical responsibility. J Am Vet Med Assoc. (2015) 246:16–8. 25654818

[78] Ünsal Adaca A Başagaç Gül RT. First experimental study in turkey teaches veterinary students how to break bad news. J Vet Med Educ. (2020) 47:720–7. 10.3138/jvme.2019-0030 32053055

[79] Chew-Graham CA Rogers A Yassin N. “I wouldn't want it on my CV or their records”: medical students' experiences of help-seeking for mental health problems. Med Educ. (2003) 37:873–80. 10.1046/j.1365-2923.2003.01627.x 12974841

[80] Knipe D Maughan C Gilbert J Dymock D Moran P Gunnell D. Mental health in medical, dentistry and veterinary students: cross-sectional online survey. BJPsych Open. (2018) 4:441–6. 10.1192/bjo.2018.61 30450222 PMC6235980

[81] Adam K Baillie S Rushton J. Retaining vets in farm animal practice: a cross-sectional study. Vet Rec. (2015) 176:655. 10.1136/vr.103170 26002092

[82] Adam KE Baillie S Rushton J. “Clients Outdoors Animals”: retaining vets in UK farm animal practice-thematic analysis of free-text survey responses. Vet Rec. (2019) 184:121. 10.1136/vr.105066 30455193

[83] Bell M Cake M Mansfield C. Success in career transitions in veterinary practice: perspectives of employers and their employees. Vet Rec. (2019) 185:232. 10.1136/vr.105133 31256013

[84] Villarroel A McDonald SR Walker WL Kaiser L Dewell RD Dewell GA. survey of reasons why veterinarians leave rural veterinary practice in the United States. J Am Vet Med Assoc. (2010) 236:859–67. 10.2460/javma.236.8.859 20392181

[85] Gerrity MS. Interventions to improve physicians' well-being and patient care: a commentary. Soc Sci Med. (2001) 52:223–5. 10.1016/S0277-9536(00)00222-7 11144778

[86] Moffett JE Bartram DJ. Veterinary Students' Perspectives on Resilience and Resilience-Building Strategies. J Vet Med Educ. (2017) 44:116–24. 10.3138/jvme.0216-046R1 28206832

[87] Powis D. Selecting medical students: An unresolved challenge. Med Teach. (2015) 37:252–60. 10.3109/0142159X.2014.993600 25532428

[88] Tötemeyer S. Selecting the right students. Vet Rec. (2013) 173:366–7. 10.1136/vr.f6214 24141162

[89] Perret JL Best CO Coe JB Greer AL Khosa DK Jones-Bitton A. Association of demographic, career, and lifestyle factors with resilience and association of resilience with mental health outcomes in veterinarians in Canada. J Am Vet Med Assoc. (2020) 257:1057–68. 10.2460/javma.2020.257.10.1057 33135980

[90] Zeijen MEL Brenninkmeijer V Peeters MCW Mastenbroek NJJM. Exploring the role of personal demands in the health-impairment process of the job demands-resources model: a study among master students. Int J Environ Res Public Health. (2021) 18:E632. 10.3390/ijerph18020632 33451042 PMC7828545

[91] Bakker AB Demerouti E. The job demands-resources model: State of the art. J Managerial Psych. (2007) 22:309–28. 10.1108/0268394071073311531861812

[92] Gordon HJ Demerouti E Le Blanc PM Bakker AB Bipp T Verhagen MAMT. Individual job redesign: job crafting interventions in healthcare. J Vocat Behav. (2018) 104:98–114. 10.1016/j.jvb.2017.07.002

[93] Lambert NM Stillman TF Hicks JA Kamble S Baumeister RF Fincham FD. To belong is to matter: Sense of belonging enhances meaning in life. Pers Soc Psychol Bull. (2013) 39:1418–27. 10.1177/0146167213499186 23950557

[94] Kanter RM. Some effects of proportions on group life: skewed sex ratios and responses to token women. Am J Sociol. (1977) 82:965–90. 10.1086/226425

[95] Torchia M Calabrò A Huse M. Women directors on corporate boards: from tokenism to critical mass. J Bus Ethics. (2011) 102:299–317. 10.1007/s10551-011-0815-z

[96] Singh B Winkel DE Selvarajan TT. Managing diversity at work: does psychological safety hold the key to racial differences in employee performance? J Occup Organ Psychol. (2013) 86:242–63. 10.1111/joop.12015

[97] Devine PG Forscher PS Austin AJ Cox WTL. Long-term reduction in implicit race bias: a prejudice habit-breaking intervention. J Exp Soc Psychol. (2012) 48:1267–78. 10.1016/j.jesp.2012.06.003 23524616 PMC3603687

[98] Horwitz IB Sonilal M Horwitz SK. Improving health care quality through culturally competent physicians: leadership and organizational diversity training. JHL. (2011) 3:29–40. 10.2147/JHL.S15620

[99] Kalra VS Abel P Esmail A. Developing leadership interventions for black and minority ethnic staff: A case study of the National Health Service (NHS) in the UK. J Health Organ Manag. (2009) 23:103–18. 10.1108/14777260910942588 19455881

[100] Sarfo-Annin JK. Ethnic inclusion in medicine: The ineffectiveness of the ‘Black, Asian and Minority Ethnic' metric to measure progress. BJGP Open. (2020) 4:BJGPO.2020.0155 10.3399/BJGPO.2020.0155 33234517 PMC7880191

[101] Aulivola B Mitchell EL Rowe VL Smeds MR Abramowitz S Amankwah KS. Ensuring equity, diversity, and inclusion in the society for vascular surgery: a report of the society for vascular surgery task force on equity, diversity, and inclusion. J Vasc Surg. (2021) 73:745–56.e6. 10.1016/j.jvs.2020.11.049 33333145

[102] Wilbur K Snyder CR Essary A Reddy S Will KK Saxon M. Developing workforce diversity in the health professions: a social justice perspective. Healts Prof Educ. (2020) 6:2-222–9 10.1016/j.hpe.2020.01.002

[103] Panagioti M Panagopoulou E Bower P Lewith G Kontopantelis E Chew-Graham C. Controlled interventions to reduce burnout in physicians: a systematic review and meta-analysis. JAMA Intern Med. (2017) 177:195–205. 10.1001/jamainternmed.2016.7674 27918798

[104] Eysenbach G. Improving the quality of web surveys: the Checklist for Reporting Results of Internet E-Surveys (CHERRIES). J Med Internet Res. (2004) 6:e34. 10.2196/jmir.6.3.e34 15471760 PMC1550605

[105] von Elm E Altman DG Egger M Pocock SJ Gøtzsche PC Vandenbroucke JP. STROBE Initiative. The Strengthening the Reporting of Observational Studies in Epidemiology (STROBE) statement: guidelines for reporting observational studies. J Clin Epidemiol. (2008) 61:344–9. 10.1016/j.jclinepi.2007.11.008 18313558

