# Supplementary material for: Mental well-being and diversity, equity, and inclusiveness in the veterinary profession: Pathways to a more resilient profession
Source: Front Vet Sci. 2022 Jul 29;9:888189. doi: 10.3389/fvets.2022.888189 (PMC9372717; doi:10.3389/fvets.2022.888189)
Supplement: Supplementary Table S1 — STROBE Statement—Checklist of items that should be included in reports of cross-sectional studies. [file Data_Sheet_1.zip › Data_Sheet_1/Table S1.DOCX]

| **Table S1. STROBE Statement—Checklist of items that should be included in reports of cross-sectional studies, adopted from (104)** | | |  |
| --- | --- | --- | --- |
|  | **Item**  **No** | **Recommendation** | **Page**  **No** |
| Title and Abstract | 1 | (a) Indicate the study’s design with a commonly used term in the title or the abstract | 1 |
|  |  | (b) Provide in the abstract an informative and balanced summary of what was done and what was found | 1 |
|  |  | **Introduction** |  |
| Background/Rationale | 2 | Explain the scientific background and rationale for the investigation being reported | 1-2 |
| Objectives | 3 | State specific objectives, including any prespecified hypotheses | 2 |
|  |  | **Methods** |  |
| Study design | 4 | Present key elements of study design early in the paper | 2 |
| Setting | 5 | Describe the setting, locations, and relevant dates, including periods of recruitment, exposure, follow-up, and data collection | 2 |
| Participants | 6 | (a) Give the eligibility criteria, and the sources and methods of selection of participants | 3 |
| Variables | 7 | Clearly define all outcomes, exposures, predictors, potential confounders, and effect modifiers. Give diagnostic criteria, if applicable | N/A |
| Data  Sources/Measurement | 8 * | For each variable of interest, give sources of data and details of methods of assessment (measurement). Describe comparability of assessment methods  if there is more than one group | N/A |
| Bias | 9 | Describe any efforts to address potential sources of bias | N/A |
| Study Sze | 10 | Explain how the study size was arrived at | 3/4 |
| Quantitative Variables | 11 | Explain how quantitative variables were handled in the analyses. If applicable, describe which groupings were chosen and why | 3 |
|  |  | (a) Describe all statistical methods, including those used to control for confounding | 3 |
|  |  | (b) Describe any methods used to examine subgroups and interactions | N/A |
| Statistical Methods | 12 | (c) Explain how missing data were addressed | N/A |
|  |  | (d) If applicable, describe analytical methods taking account of sampling strategy | N/A |
|  |  | (e) Describe any sensitivity analyses | N/A |
|  |  | **Results** |  |
|  |  | (a) Report numbers of individuals at each stage of study—eg numbers potentially eligible, examined for eligibility, confirmed eligible, included in the  study, completing follow-up, and analyzed | 3/4 |
| Participants | 13 * | (b) Give reasons for non-participation at each stage | N/A |
|  |  | (c) Consider use of a flow diagram | Fig. 2 |
| Descriptive Data | 14 * | (a) Give characteristics of study participants (eg demographic, clinical, social) and information on exposures and potential confounders | Tab. 1 |
|  |  | (b) Indicate number of participants with missing data for each variable of interest | N/A |
| Outcome Data | 15* | Report numbers of outcome events or summary measures | 4-6 |
|  |  | (a) Give unadjusted estimates and, if applicable, confounder-adjusted estimates and their precision (eg, 95% confidence interval). Make clear which  confounders were adjusted for and why they were included | N/A |
| Main Results | 16 | (b) Report category boundaries when continuous variables were categorized | N/A |
|  |  | (c) If relevant, consider translating estimates of relative risk into absolute risk for a meaningful time period | N/A |
| Other Analyses | 17 | Report other analyses done—eg analyses of subgroups and interactions, and sensitivity analyses | N/A |
|  |  | **Discussion** |  |

| Key Results | 18 | Summarize key results with reference to study objectives | 7 |
| --- | --- | --- | --- |
| Limitations | 19 | Discuss limitations of the study, taking into account sources of potential bias or imprecision. Discuss both direction and magnitude of any potential bias | 11 |
| Interpretation | 20 | Give a cautious overall interpretation of results considering objectives, limitations, multiplicity of analyses, results from similar studies, and other  relevant evidence | 8-10 |
| Generalizability | 21 | Discuss the generalizability (external validity) of the study results | 11 |
|  |  | **Other information** |  |
| Funding | 22 | Give the source of funding and the role of the funders for the present study and, if applicable, for the original study on which the present article is  based | 12 |

* Give information separately for exposed and unexposed groups.
