# Supplementary material for: Mental well-being and diversity, equity, and inclusiveness in the veterinary profession: Pathways to a more resilient profession
Source: Front Vet Sci. 2022 Jul 29;9:888189. doi: 10.3389/fvets.2022.888189 (PMC9372717; doi:10.3389/fvets.2022.888189)
Supplement: Supplementary Table S1 — STROBE Statement—Checklist of items that should be included in reports of cross-sectional studies. [file Data_Sheet_1.zip › Data_Sheet_1/Table S3.DOCX]

| **Title** | **FVE/WVA/Zoetis Survey on veterinary Mental Health, Inclusiveness, Equity and Diversity** | | | | | | | | | | |
| --- | --- | --- | --- | --- | --- | --- | --- | --- | --- | --- | --- |
| **Introduction** | The 2018 VetSurvey on the demography of the veterinary profession in Europe indicated that many veterinarians face important stress at work. Indeed, in many countries worldwide, there is an increasing concern around the mental health of veterinarians. The COVID-19 pandemic further increased the mental burden on the veterinarian. Recognising these developments, several veterinary organisations, institutions, and companies have taken action to implement measures for improving veterinary mental health and wellbeing.  Recently, growing attention is given to increase the diversity and inclusiveness of our profession in respect to age, disability, race, ethnicity, gender, sexual identity, sexual preference, religion, personality, lifestyle, personal decisions, etc.    With this short survey, the Federation of Veterinarians of Europe (FVE), in collaboration with the World Veterinary Association (WVA), and Zoetis, aims to collect information on how veterinary associations, societies and companies in Europe and worldwide ensure veterinary well-being and how they promote diversity and inclusiveness within our profession. We are hoping to get a contribution at country/organisation/company level, i.e. one answer per institution/organisation.  The results of this survey will be used to write a report about activities in respect to veterinary well-being, diversity, and inclusiveness throughout the world. Before publication, results of the survey will be aggregated per region/country, and all personal data will be anonymized.  With these results, we hope to foster and stimulate reflections on improvement of mental health support systems for veterinarians, on actions to support diversity and inclusiveness’ awareness, as well as to facilitate exchange of good practices. The results will also be used by the FVE Board for further consideration on policies and actions that need to be taken, for example the need for the establishment of a dedicated working group to work on this issue.  Data will be handled anonymously and in line with the EU GDPR rules.  For questions: Florentine Timmenga, intern FVE (info@fve.org) | | | | | | | | | | |
| **Question** | **Answer type** | **Answer options** | | | | | | | | | |
| 1 a. Name of your organisation/institution/company | Short paragraph |  | | | | | | | | | |
| 1 b. Country of residence of your organisation/institution/company/practice type | Short paragraph |  | | | | | | | | | |
| 1 c. Your name as representative person replying for your organisation/institution/company/practice type | Short paragraph |  | | | | | | | | | |
| 1 d. Your position within the organisation/institution/company/practice | Short paragraph |  | | | | | | | | | |
| 2 a. Is mental well-being (stress, burn-out, depression, compassion fatigue, suicidal ideation, etc.) explicitly designated as a key priority area with a dedicated budget in your organisation/company? | Multiple Choice | Yes | | | Partially | | | | No | | |
| 2 b. If yes, how does your organisation/company ensure implementation? (multiple answers are possible) | Multiple Choice | Part of the veterinary code of conduct/ mission statement/etc | | By having a dedicated body/  committee/  department, ombudsperson | | | By having rules in place to investigate and promote well-being | | | Other | |
| 2 c. You may provide more information here | Short paragraph |  | | | | | | | | | |
| 3 a. Is diversity, equity and inclusiveness (age, disability, race, ethnicity, gender, sexual identity, sexual preference, religion, personality, lifestyle, etc) explicitly designated as a key priority area with a designated budget in your organisation/company? | Multiple Choice | Yes | | | Partially | | | | No | | |
| 3 b. If yes, how does your organisation/company ensure implementation? (multiple answers are possible) | Multiple Choice | Part of the veterinary code of conduct/  mission statement/  etc. | By having a dedicated body/ committee/ department, ombudsperson | | | By having rules in place to investigate and promote DEI | | By having quota for leadership positions | | | Publishing data on DEI statistic or other relevant data |
| 3 c. You may provide more information here | Short paragraph |  | | | | | | | | | |
| 4 a. Does your organisation/company have committees/coaches/education and training to increase and improve mental well-being? | Multiple Choice | Yes | | | Not yet, but planning | | | | No | | |
| 4 b. If yes, please give examples of projects/activities organised e.g. ombudspersons, training courses, helpline, communications campaigns, webinars to raise awareness, etc | Long paragraph |  | | | | | | | | | |
| 5 a. Does your organisation/company have committees/coaches/ education and training to increase and improve diversity, equity, and inclusiveness (age, disability, race, ethnicity, equity, gender, sexual identity, sexual preference, religion, etc) ? | Multiple Choice | Yes | | | Not yet, but planning | | | | No | | |
| 5 b. If yes, please give examples of projects/activities organised | Long paragraph |  | | | | | | | | | |
| 6. If you have projects or activities, can you share any weblinks, documents or publications with us referring to the activities in your organisation / company? Please send them to stagiaire@fve.org | Long paragraph |  | | | | | | | | | |
| 6 a. If you have projects or activities, what do you think was the impact of the activities taken by your organisation/company regrading mental wellbeing? (Please score 0-5, where 0 is having no impact and 5 represents a very positive impact,). Only score for the activities you have in your organisation/company. | Multiple Choice Grid |  | | | | | | | | | |
|  | Part of the Mission/Vision/Internal rules | 1 | 2 | | | 3 | | 4 | | | 5 |
|  | Dedicated working group or committee | 1 | 2 | | | 3 | | 4 | | | 5 |
|  | Ombudsperson | 1 | 2 | | | 3 | | 4 | | | 5 |
|  | Helpline | 1 | 2 | | | 3 | | 4 | | | 5 |
|  | Awareness campaigns | 1 | 2 | | | 3 | | 4 | | | 5 |
|  | Training/webinars | 1 | 2 | | | 3 | | 4 | | | 5 |
|  | Quota to ensure diversity in management functions | 1 | 2 | | | 3 | | 4 | | | 5 |
|  | Regular investigation of well-being and non-discrimination of employees | 1 | 2 | | | 3 | | 4 | | | 5 |
|  | Description of rules/sanctions foreseen in cases of noncompliance and of responsibilities in the internal rules | 1 | 2 | | | 3 | | 4 | | | 5 |
|  | Other | 1 | 2 | | | 3 | | 4 | | | 5 |
| 6 b. If you have projects or activities, what do you think was the impact of the activities taken by your organisation/company regrading diversity and inclusiveness? (Please score 0-5, where 0 is having no impact and 5 represents a very important impact,). Only score for the activities you have in your organisation/company. | Multiple Choice Grid |  | | | | | | | | | |
|  | Part of the Mission/Vision/Internal rules | 1 | 2 | | | 3 | | 4 | | | 5 |
|  | Dedicated working group or committee | 1 | 2 | | | 3 | | 4 | | | 5 |
|  | Ombudsperson | 1 | 2 | | | 3 | | 4 | | | 5 |
|  | Helpline | 1 | 2 | | | 3 | | 4 | | | 5 |
|  | Awareness campaigns | 1 | 2 | | | 3 | | 4 | | | 5 |
|  | Training/webinars | 1 | 2 | | | 3 | | 4 | | | 5 |
|  | Quota to ensure diversity in management functions | 1 | 2 | | | 3 | | 4 | | | 5 |
|  | Regular investigation of well-being and non-discrimination of employees | 1 | 2 | | | 3 | | 4 | | | 5 |
|  | Description of rules/sanctions foreseen in cases of noncompliance and of responsibilities in the internal rules | 1 | 2 | | | 3 | | 4 | | | 5 |
|  | Other | 1 | 2 | | | 3 | | 4 | | | 5 |
| 6 c. If you have other indicators in place to measure the outcomes of your activities please describe here (open comment) | Long paragraph |  | | | | | | | | | |
| 7. Are there national surveys, publications or reports about mental health, diversity, equity and inclusiveness for the veterinary profession in your country? | Multiple Choice | Yes | | | | | No | | | | |
| 7 a. We would love to get more reports, publications and surveys on mental health, diversity and inclusiveness from your country or region. Please send them to stagiaire@fve.org | Long paragraph |  | | | | | | | | | |
| 8. Would you like to make any additional comments/information to us regarding the above-mentioned topics? | Long paragraph |  | | | | | | | | | |
| 9. Would you believe sharing of global best practice on these topics would be valuable? | Multiple Choice | Yes | | | | | No | | | | |
| 10. Do you agree to be contacted for more information/ sharing the results of the project when published? If yes please leave email below. | Short paragraph |  | | | | | | | | | |
