# Supplementary material for: Mental well-being and diversity, equity, and inclusiveness in the veterinary profession: Pathways to a more resilient profession
Source: Front Vet Sci. 2022 Jul 29;9:888189. doi: 10.3389/fvets.2022.888189 (PMC9372717; doi:10.3389/fvets.2022.888189)
Supplement: Supplementary Table S1 — STROBE Statement—Checklist of items that should be included in reports of cross-sectional studies. [file Data_Sheet_1.zip › Data_Sheet_1/Table S4.docx]

Supplementary Table S4. Interviewed organizations and questions for interviewees who were selected based on the online survey of those who had support programs available

| Interviewee and affiliation | *Questions* |
| --- | --- |
| Interviewee 1 - MMI & Vetlife  Interviewee 2 - MSD  Interviewee 3 - Kenyan Veterinary Association  Interviewee 4 - Colegio de médicos veterinarios de Costa Rica  Interviewee 5 - HVA  Interviewee 6 - FECAVA  Interviewee 7 - IVSA  Interviewee 8 - SAVA & University of Pretoria  Interviewee 9 - AVMA  Interviewee 10 - VCI  Interviewee 11 - LGBTQI+ Germany  Interviewee 12 - Swedish Veterinary Association  Interviewee 13 - Utrecht University & KNMvD  Interviewee 14 – Zoetis | *What is the geographical coverage of the program?* |
|  | *What are the results and how is the impact in the different countries? Are there reginal/national differences?* |
|  | *Are there differences in MWB/DEI issues between the different veterinary professional groups?* |
