# Supplementary material for: Mental well-being and diversity, equity, and inclusiveness in the veterinary profession: Pathways to a more resilient profession
Source: Front Vet Sci. 2022 Jul 29;9:888189. doi: 10.3389/fvets.2022.888189 (PMC9372717; doi:10.3389/fvets.2022.888189)
Supplement: Supplementary Table S1 — STROBE Statement—Checklist of items that should be included in reports of cross-sectional studies. [file Data_Sheet_1.zip › Data_Sheet_1/Table S5.docx]

| **FVE/ZOETIS VETERINARY WELL-BEING WEBINAR** | **FVE/ZOETIS VETERINARY DIVERSITY, EQUITY & INCLUSIVENESS WEBINAR** |
| --- | --- |
| **PROGRAMME** | **PROGRAMME** |
| Word of welcome  RENS VAN DOBBENBURGH (FVE PRESIDENT) & CHRISTINE JENKINS (ZOETIS) | Word of welcome  RENS VAN DOBBENBURGH (FVE PRESIDENT) & DANIEL EDGE (ZOETIS) |
| Quiz & short introduction FVE/WVA/ZOETIS MENTAL HEALTH & DEI SURVEY 2021 | Quiz & short introduction FVE/WVA/ZOETIS MENTAL HEALTH & DEI SURVEY 2021 |
| **SPEAKERS** | **SPEAKERS** |
| NICOLE MASTENBROEK (UTRECHT UNIVERSITY): The art of staying engaged; how to support sustainable employability of young vets | FLORENTINE TIMMENGA (FVE): preliminary results FVE/WVA/ZOETIS MENTAL HEALTH & DEI SURVEY 2021 |
| JOSEPH HAHN (MSD): Results of the MERCK ANIMAL HEALTH, AVMA & BRAKKE study on veterinary wellbeing | MIA CARY (PRIDEVMC): PRIDEVMC – Who we are & why we exist |
| FLORENTINE TIMMENGA (FVE): Preliminary results FVE/WVA/ZOETIS MENTAL HEALTH & DEI SURVEY 2021 | NAVEESHA SHERGILL (IVSA): IVSA report on DEI & discrimination survey in the veterinary community |
| AIDEEN HEALY (VETERINARY COUNCIL OF IRELAND): SAFEVET HANDBOOK | TOM DOYLE (BVLGBT+ ASSOCIATION): British veterinary LGBT+: Its history, raison d’être and future |
| **STATEMENT-DISCUSSION WITH EXPERTPANEL** | **STATEMENT-DISCUSSION WITH EXPERTPANEL** |
| CHRISTINE JENKINS (ZOETIS) | DANIEL EDGE (ZOETIS) |
| LIDEWIJ WIERSMA (EBVS) | WARD DE SPIEGELAERE (Ghent University) |
| **Statements** | **Statements** |
| What have you seen that is working, in terms of supporting mental health and wellbeing amongst the veterinary team? Why do you think it’s effective? | Discussion of the quiz-question ‘Is DEI an easy to talk about topic? |
| How do we better communicate the skills required to be a successful veterinarian to potential vet students before starting and better support them through veterinary school? | Do DEI support programs need to focus mainly on the leading generation or the future generation? |
| How can veterinary associations assist employers in their efforts to provide a mentally healthy workplace with the demands of clients and the reality of the job? | Which support programs are most effective in improving DEI in the veterinary profession? E.g. part of the veterinary code of conduct, having a dedicated body/committee, awareness campaigns, webinars/seminars, having rules in place, regular investigation of DEI, having quota for certain position? |
| If we had to focus our efforts in one area, what do you think it should be? |  |
